# Case report: A *de novo* RASopathy-causing *SHOC2* variant in a Chinese girl with noonan syndrome-like with loose anagen hair

**DOI:** 10.3389/fgene.2022.1040124

**Published:** 2022-12-09

**Authors:** Qingming Wang, Shuangxi Cheng, Youqing Fu, Haiming Yuan

**Affiliations:** ^1^ Dongguan Maternal and Child Healthcare Hospital, Dongguan, China; ^2^ Dongguan Institute of Reproductive and Genetic Research, Dongguan, China

**Keywords:** SHOC2, Noonan syndrome-like with loose anagen hair (NS/LAH), short stature, growth hormone (GH), growth hormone deficiency (GHD)

## Abstract

Pathogenic variants in the RASopathy-causing *SHOC2* gene have been suggested to cause Noonan syndrome-like with loose anagen hair (NS/LAH). This condition is characterized by facial features resembling Noonan syndrome (NS), short stature, growth hormone deficiency (GHD), cognitive deficits, cardiac defects, and ectodermal abnormalities, including easily pluckable, sparse, thin, slow-growing hair, hyperpigmented skin and hypernasal voice. The mutation spectrum of *SHOC2* is narrow, and only 8 pathogenic variants have been identified. Here, we report a 5-year-3-month-old Chinese female who displays characteristics typical of NS and has normal neurodevelopment. Trio-based whole-exome sequencing (WES) revealed a *de novo* variant (c.1231A>G, p.Thr411Ala) in *SHOC2*. This variant has been recently reported in one subject in the literature who displayed facial features typical of NS and also presented with significant speech delays, moderate intellectual disabilities, epilepsy, bilateral sensorineural deafness and renal dysplasia. The differential phenotypes between these subjects deserve to be further investigated. Next, we reviewed the clinical pictures of NS/LAH and noticed that a recurrent *SHOC2* Ser2Gly variant was more likely to result in delayed neurodevelopment and short stature, compared to other *SHOC2* variants. And growth hormone (GH) therapy could improve height prognosis. It was noticed that the slight sleep problems and friendly and relatively mature personality observed in our patient may be a novel phenotype of NS/LAH. Our study reconfirms the pathogenic nature of the *SHOC2* Thr411Ala variant. It also provides insights into the genotype-phenotype relationship in NS/LAH and a foundation for its genetic counseling, diagnosis and treatment.

## Introduction

Noonan syndrome-like with loose anagen hair (NS/LAH) (MIM:607721) is a very rare autosomal dominant RASopathy. This condition was characterized by facial anomalies similar to those observed in NS, short stature frequently with growth hormone deficiency (GHD), intellectual disability, ectodermal abnormalities, including easily pluckable, sparse, thin slow-growing hair, recurrent eczema and ichthyosis, dark pigmented skin, hypernasal voice and cardiac defects (especially dysplasia of the mitral valve and septal defects) (Cordeddu et al., 2009; Hoban et al., 2012; Capalbo et al., 2012b; Gripp et al., 2013; Baldassarre et al., 2014; Hannig et al., 2014; Motta et al., 2019; Motta et al., 2022). Currently, only 8 pathogenic *SHOC2* variants have been reported to cause NS/LAH through gain-of-function mechanisms (Cordeddu et al., 2009; Hannig et al., 2014; Young et al., 2018; Motta et al., 2019; Motta et al., 2022). SHOC2 is a scaffold protein comprising an N-terminal lysine-rich region and subsequent 19 leucine-rich repeats (LRRs) that plays a crucial role in the activation of the ERK1 (MAPK3; 601795)/ERK2 (MAPK1; 176948) signaling pathway (Cordeddu et al., 2009; Rodriguez-Viciana et al., 2006; Matsunaga-Udagawa et al., 2010). Among all variants, a recurrent activating mutation in *SHOC2* (p.Ser2Gly) has been frequently reported in NS/LAH patients with homogeneous clinical manifestations (Cordeddu et al., 2009; Gripp et al., 2013). The remaining pathogenic variants in *SHOC2* might cause a milder phenotype (Hannig et al., 2014; Motta et al., 2019; Motta et al., 2022). Remarkably, a *de novo SHOC2* variant (Thr411Ala) was previously reported in Subject 3 in the literature. This subject presented with facial features typical of NS and also displayed severe phenotypes including neurodevelopmental delay, epilepsy, bilateral sensorineural deafness and renal dysplasia (Motta et al., 2022). Here, we again identified the *de novo SHOC2* variant (c.1231A>G, p.Thr411Ala) in a 5-year-3-month-old girl who only displayed characteristics typical of NS/LAH. The report reconfirms the pathogenic nature of the variant. Whereas, the differential phenotypes between the two subjects deserve to be further investigated. Next, we systematically reviewed the clinical characteristics of individuals with NS/LAH.

## Materials and methods

### Ethical compliance

This study was approved by the Ethics Committee of Dongguan Maternal and Child Healthcare Hospital (DMCH 2020-6). Written informed consent was obtained from the legal guardians for the publication of any potentially identifiable images or data included in this article.

### Trio-based whole-exome sequencing (WES)

Trio-based whole-exome sequencing (Illumina, San Diego, CA, United States) was performed for the family. Sequencing was performed with an Illumina NovaSeq 6,000 (Illumina, San Diego, CA, United States). The bcl2fastq2 Conversion Software (v2.20) was employed for extracting Fastq files, and all reads were mapped to the human genome (GRCh37/hg19) by using BWA (v0.2.10) with default parameters. The Genome Analysis Toolkit (GATK; v.3.7) HaplotypeCaller was applied for identifying variants. The aligned reads were visualized by using the Integrated Genome Viewer (IGV). Common variants were filtered based on their frequencies in the databases of the Exome Sequencing Project (https://esp.gs.washington.edu), the Exome Aggregation Consortium (ExAC) (http://exac.broadinstitute.org), or 1,000G (http://www.1000genomes.org), and our internal database. The suspected variant was verified by Sanger sequencing. The pathogenicity of the sequence variants was interpreted according to ACMG/AMP guidelines (Richards et al., 2015).

## Results

### Clinical report

The proband was the first female child of unrelated Chinese parents. Her 2-year-old younger sister was apparently healthy. Her birth measurements were normal: length 49 cm, weight 2.85 kg and head circumference 34.5 cm.

**FIGURE 1:**
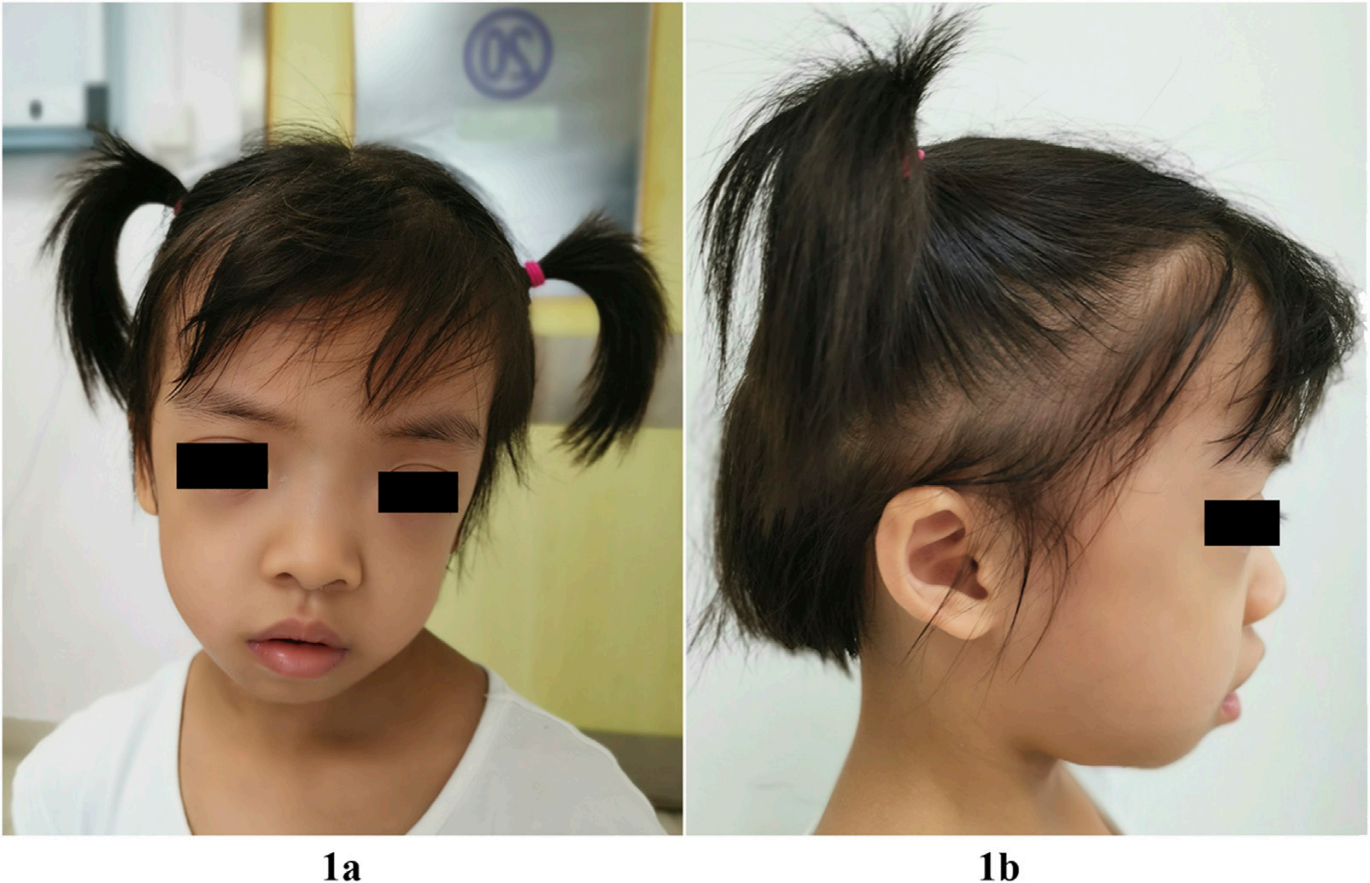
Facial features of our patient with the *SHOC2* variant (p.Thr411Ala) at 5-year-3-month of age. Note relatively macrocephaly, broad and square forehead, frontal bossing, hypertelorism, long eyelashes, unilateral left ptosis, downslanting palpebral fissures **(1A)**, low-set posteriorly angulated ears, and overfolded pinnae **(1B)**, sparse, thin, slow-growing hair, but not loose **(1A,1B)**.

Failure to thrive prompted hospitalization at the age of 4 years and 2 months. At this time, her height was 91.5 cm (<-3.3 SD), her weight was 12 kg (-3 SD) and her head circumference was 50.8 cm (1 SD). She had normal motor development. She raised her head at 3 months, sat alone at 7 months and independently walked at 1 year 2 months. She had craniofacial anomalies characterized by relatively macrocephaly, hypertelorism, long eyelashes, unilateral left ptosis, downslanting palpebral fissures, low-set posteriorly angulated ears, overfolded pinnae, teeth dysplasia, and hair anomalies, including sparse, thin, slow-growing hair that was not loose. She had mild frontal bossing, and her forehead appeared broad and square (Figure 1). Mild aortic and tricuspid regurgitation was revealed by echocardiography. Deep palmar lines and hypotonia were noted. No feeding difficulties were recorded, but she was a picky eater. She displayed slight sleep problems. She was friendly and seemed to have a relatively mature personality beyond her age. She had recurrent eczema and darkly pigmented skin. Her neck, chest, and back were non-dysmorphic. Her bone age was delayed by 2 years at chronological age 4 years 2 months. An endocrinological evaluation revealed partial GHD for IGF1 (23.0 ng/ml, n: 41.3-229.2) and IGFBP-3 (1.78 ng/ml, n: 1.0-4.7). GH therapy (50 μg/kg/day) was initiated at the age of 4 years and 5 months with height 92.0 cm (−3.5 SD) and weight 12.5 Kg (−2.8 SD). This resulted in increased growth velocity, with a height of 101 cm (−2.5 SD) and a weight of 14.5 kg (− 2.0 SD) at 5 years and 3 months of age. IGF1 level was remarkably improved (IGF1: 101 ng/ml). No adverse events occurred. She displayed normal cognitive and language abilities through professional neurological assessments. Brain magnetic resonance imaging was normal at this age. The patient was clinically diagnosed with NS assessed by experienced clinical specialists.

### Genetic analysis

**FIGURE 2:**
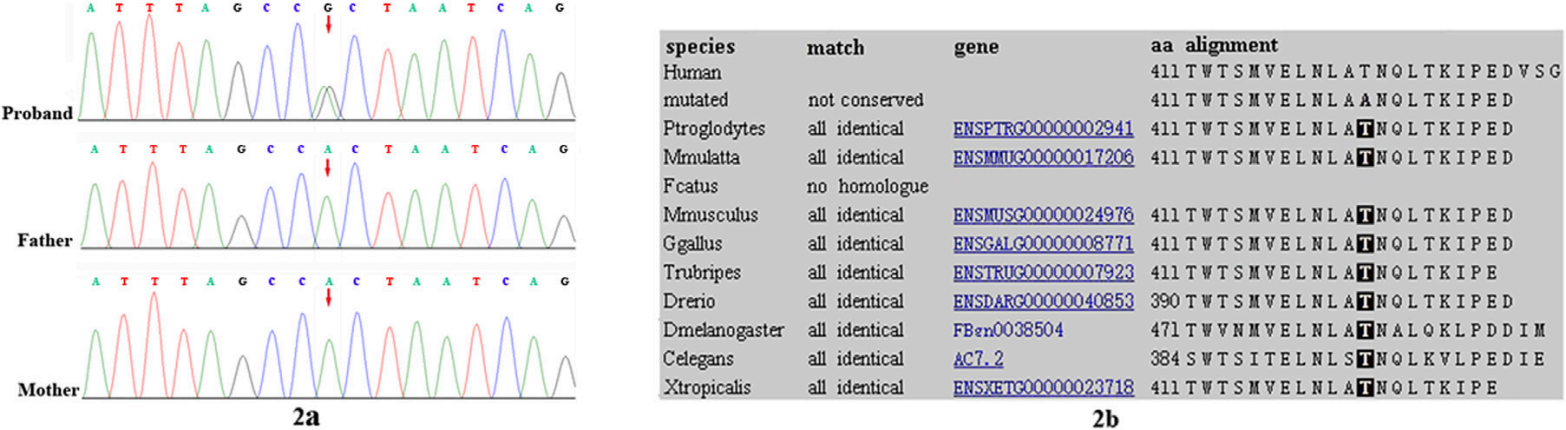
Sanger sequencing results for (A) our patient, and the patient’s (B) mother and (C) father. The analysis demonstrated the presence of a missense *SHOC2* variant (c.1231A>G, p.Thr411Ala; red arrow) in our patient and the absence of the variant in her parents **(2A)**. Location of patient’s *SHOC2* missense variant (p. Thr411Ala) on a highly conserved multi-species alignment of SHOC2 protein sequence, shaded area stands for the residue Thr411 **(2B)**.

Trio-based WES identified a heterozygous variant, c.1231A>G, p.Thr411Ala, in the *SHOC2* gene in our patient. The variant was validated by bidirectional Sanger sequencing, which also demonstrated its *de novo* event (Figure 2A) (PS2). The Thr411 residue is located in LRR14 and is highly conserved among different species (PM1) (Figure 2B). This variant had not been reported in the public databases (ExAC, gnomAD, 1000 Genomes Project) or our internal database (PM2). This variant was predicted to have a deleterious effect on the gene product by multiple *in silico* prediction tools (SIFT, MutationTaster, PolyPhen-2). In addition, the patient’s phenotypes were highly consistent with those of NS/LAH (PP4), and WES also excluded other possible known genetic causes. Furthermore, this variant has been previously reported to be a *de novo* event in Subject 3 with NS-like facial features (Motta et al., 2022). Thus, this variant was categorized as clinically pathogenic according to ACMG/AMP guidelines (PS2+PM1+PM2+PP3+PP4) (PS: pathogenic strong; PM: pathogenic moderate; PP: pathogenic supporting).

## Discussion

The *SHOC2* gene was infrequently reported in NS patients. Currently, only 8 pathogenic variants in *SHOC2* were identified to cause NS/LAH through gain-of-function mechanisms (Cordeddu et al., 2009; Hannig et al., 2014; Young et al., 2018; Motta et al., 2019; Motta et al., 2022). Here, we reported a 5-year-3-month-old Chinese female who has distinctive facial features (relatively macrocephaly, hypertelorism, long eyelashes, unilateral left ptosis, downslanting palpebral fissures, low-set posteriorly angulated ears, overfolded pinnae, teeth dysplasia, and sparse, thin, slow-growing hair), short stature, partial GHD, recurrent eczema and darkly pigmented skin, deep palmar lines and hypotonia, but she demonstrated normal cognitive and language development. WES identified a *de novo* missense variant (p.Thr411Ala) in *SHOC2*, and WES excluded other possible causal variants**.** This variant was classified as clinically pathogenic and was likely to be responsible for our patient’s clinical phenotypes.

Next, we reviewed the clinical features of individuals with pathogenic *SHOC2* variants to comprehensively profile the condition (Table 1). All patients had the typical facial gestalt and sparse eyebrows, and easily pluckable, sparse, thin, slow-growing hair of NS/LAH. Most patients had short/broad neck (7/9), motor delay (6/9) and cardiac defects (6/9). The other phenotypes include short stature (4/9), darkly pigmented skin (4/9), recurrent eczema or ichthyosis (4/9) and feeding difficulties (3/9). Furthermore, the features that hypotonia and deep palmar lines observed in our patient and Subject 1 may be uncommon characteristics of NS/LAH. A recurrent activating mutation in *SHOC2* (p.Ser2Gly) has been frequently reported in NS/LAH patients with a higher prevalence of speech delays, cognitive deficits and abnormal intracranial structures (Cordeddu et al., 2009; Capalbo et al., 2012a; Gripp et al., 2013; Baldassarre et al., 2014; Li et al., 2019; Lores, 2020). Pathogenic variants other than p.Ser2Gly cause milder phenotypes with normal or mildly impaired speech or cognitive development (Hannig et al., 2014; Motta et al., 2022). Severe short stature is frequently reported in *SHOC2* Ser2Gly variant patients who exhibit mild to moderate GHD and growth hormone insensitivity (GHI) and thus require a higher dose of GH therapy (e.g., 35–40 μg/kg/day) (Cordeddu et al., 2009; Capalbo et al., 2012a; Capalbo et al., 2012b; Mazzanti et al., 2013). Whereas, a previously reported patient with Ser2Gly variant who had severe short stature was remarkably improved by low-dose GH therapy (25 μg/kg/day) (Takasawa et al., 2015). Currently, short stature was only described in two subjects with pathogenic variants (Met173Val and Gln269Arg respectively) other than Ser2Gly, in the recently published literature. The two subjects have GHD and have good response to GH therapy (Motta et al., 2022). Our patient carrying the *SHOC2* Thr411Ala variant had obvious short stature with partial GHD. The GH therapy (50 μg/kg/day) obviously improved the linear growth of our patient, with her height reaching -2.5 SD after 11 months of treatment. These findings suggest that short stature occurred in four of eight variants, and GH therapy would be beneficial to the improvement of height.

**TABLE 1:** Overview of variants and phenotypes observed in patients with NS/LAH.

Clinical features	Ser2Gly Cordeddu et al. (2009)	Gly53Arg Motta et al. (2022)	Met173Ile Hannig et al. (2014)	Met173Val Motta et al. (2022)	Met173_Leu174delinsIlePhe Motta et al. (2022)	Gln269Arg Motta et al. (2022)		Gln269_His270delinsHisTyr Motta et al. (2019)	Thr411Ala Motta et al. (2022)	Thr411Ala Our patient
		Subject 6		Subject 2	Subject 5	Subject 1	Subject 4		Subject 3	
Segregation	*de novo*	*de novo*	*inherited from father with similar phenotypes*	*de novo*	*de novo*	*de novo*	*de novo*	*de novo*	*de novo*	*de novo*
NS-like facial features	+	+	+	+	+	+	+	+	+	+
Sparse eyebrows	+	+	+	+	+	+	+	+	+	+
Short/broad neck	+	+	−	−	+	+	+	+	+	−
easily pluckable, sparse, thin slow-growing hair	+	+	+	+	+	+	+	+	+	+
Intellectual disability	+	−	mild	mild	−	−	−	NA	moderate	−
Language delay	+	−	+	+	−	−	+	NA	+	−
Motor delay	+	−	−	+	+	+	+	NA	+	−
Short stature	+	−	−	+	−	mild	+	NA	−	+
Growth hormone deficit	+	−	−	+	-	+	+	NA	−	+
Feeding difficulties	−	−	−	+	+	−	+	NA	−	a picky eater
Darkly pigmented skin	+	−	−	+	+	−	−	−	−	+
Recurrent eczema or ichthyosis	+	−	−	−	+	−	+	−	−	+
Cardiac defects	+	+	−	−	+	−	−	+	+	+
Others	abnormal intracranial structure hypemasal voice	autoimmune thyroiditis	None	None	Constipation	hypemasal voice hypotonia deep palmar lines	−	hypospadias	epilepsy deafness renal dysplasia	sleep problems friendly and mature personality hypotonia deep palmy lines

F, female; M, male; +, present; −, absent; NA, not available.

Remarkably, in contrast to our patient with severe short stature, the *de novo SHOC2* variant (Thr411Ala) was previously identified in Subject 3 in the recently published literature, who had normal height (Motta et al., 2022). Both individuals presented with facial features typical of NS. However, Subject 3 displayed severe phenotypes. Subject 3 showed significant speech delay, moderate intellectual disabilities and epilepsy, whereas our patient had normal neurodevelopment. Furthermore, Subject 3 suffered from bilateral sensorineural deafness requiring hearing aids and unilateral renal dysplasia due to ureteral reflux and requiring left nephrectomy (Motta et al., 2022). These phenotypes are not consistent with those of NS, and our patient did not show these features. Our patient’s milder phenotypes seem to be more consistent with those caused by pathogenic variants other than p.Ser2Gly. Therefore, it is possible that there may be additional pathogenic variants or copy number variants (CNVs) contributing to these differential phenotypes observed for Subject 3, as the variant analysis for this subject was detected using the RASopathy gene panel instead of whole-exome sequencing. This significant discrepancy needs to be further investigated. It is worth mentioning that our patient displayed slight sleep problems and a friendly and relatively mature personality, which have not been depicted in previously reported NS/LAH individuals. Thus, the present case enriches the clinical features of NS/LAH.

In conclusion, the *SHOC2* Thr411Ala variant identified here further confirmed the pathogenic nature of the variant. Next, we systematically reviewed the clinical phenotypes of NS/LAH individuals. The novel phenotypes, slight sleep problems and a friendly and relatively mature personality, were observed in our patient. These findings will enrich our knowledge of the clinical characteristics, clinical management and genetic counseling of NS/LAH, which needs to be further explored.

## Data Availability

The data analyzed in this study is subject to the following licenses/restrictions: The datasets for this article are not publicly available due to concerns regarding participant/patient anonymity. Requests to access the datasets should be directed to the corresponding author. Requests to access these datasets should be directed to haimingyuan, haimingyuan@sina.cn.
